# The diagnostic performance of ss-EPI-DWI, rs-EPI-DWI (RESOLVE) and TGSE-BLADE-DWI in a model of retinal ischemia: a comparative phantom study

**DOI:** 10.1038/s41598-025-30769-w

**Published:** 2025-12-03

**Authors:** Alexander Schütze, Yannick Schröder, Shakhnaz Guseynova, Sebastian Vellmer, Eberhard Siebert, Michael Scheel

**Affiliations:** Department of Neuroradiology, Charité Universitätsmedizin Berlin, Berlin, Germany

**Keywords:** TGSE-BLADE-DWI, Central retinal artery occlusion, Retina, rs-EPI-DWI, ss-EPI-DWI, DWI-phantom, Diseases, Medical research, Neurology, Neuroscience

## Abstract

This study systematically compared the performance of ss-EPI-DWI, rs-EPI-DWI (RESOLVE), and TGSE-BLADE-DWI for detecting small ischemic lesions (10 –2 mm) using a DWI phantom to gain insights for optimization of microstructural orbital MRI for retinal ischemia diagnosis. A DWI phantom simulating ischemic lesions embedded in healthy neuronal tissue was used to quantitatively and qualitatively assess the sequences at 3T. Signal-to-noise-ratio (SNR), contrast-to-noise-ratio (CNR), relative contrast (ReCon), geometric distortion rate (GDR) and apparent diffusion coefficient (ADC) accuracy were measured, with correlation analysis examining the effect of lesion size. Qualitative analysis was performed by two neuroradiologists evaluating the images in different criteria using a 5-point Likert scale. Among all sequences rs-EPI-DWI showed the most precise ADC (*p* < 0.001), highest CNR (*p* < 0.001) and relative contrast (*p* < 0.001) and non-inferiority to ss-EPI-DWI in SNR (*p* = 0.06). Both TGSE-BLADE-DWI and rs-EPI-DWI showed less GDR and susceptibility artifacts than ss-EPI-DWI qualitatively. All sequences depicted even the 2 mm lesion qualitatively; however, lesion size most strongly affected TGSE-BLADE-DWI quantitatively. Therefore, rs-EPI-DWI and TGSE-BLADE-DWI are promising alternatives to conventional ss-EPI-DWI for microstructural orbital MRI and may prove useful for clinical diagnosis of acute retinal ischemia due to high spatial resolution with reliable ADC acquisition, low geometric distortion and reduced susceptibility artifacts.

**Supplementary Information:**

The online version contains supplementary material available at 10.1038/s41598-025-30769-w.

## Introduction

Diffusion weighted imaging (DWI) has seen significant advancements over the last two decades, emerging as a pivotal tool in clinical MRI. With improvements in gradient strengths, specialized coils, and optimized imaging sequences, DWI is now increasingly used for orbital imaging^1–3^. As DWI assesses diffusion properties of protons, it is particularly sensitive towards diffusion restrictions in extracellular water^3^, which can indicate inflammatory^4,5^, vascular^2^, or neoplastic conditions^6–8^ affecting the orbit, eye, or optic nerve. DWI was recently extended to the imaging of retinal ischemia, focusing on the central retinal artery occlusion (CRAO)^9–12^, branch retinal artery occlusion (BRAO)^13^ as well as the anterior ischemic optic neuropathy (AION)^14^ - acute vascular pathologies traditionally diagnosed by ophthalmological methods. Thus, DWI could offer valuable supplementary diagnostic information for ischemic retinal diseases.

Despite its potential, DWI faces several drawbacks in ocular MRI. Conventional single-shot-echo-planar-imaging (ss-EPI)-DWI struggles with magnetic susceptibility artifacts at air-bone-tissue interfaces^15,16^, complicating high-quality imaging of the eye near the paranasal sinuses^17,18^. Additionally, despite various compensation strategies such as the implementation of navigator echoes, pulse triggering or gradient adjustments^19,20^ sensitivity of the diffusion gradients to motion induced artifacts continues to be a challenge in several DWI sequences. Furthermore, a major issue seems to be the small size of the retina, complicating an accurate depiction of retinal pathologies even more^21,22^.

To address some of these limitations, several alternative DWI sequences have been proposed. Readout-segmented-EPI (rs-EPI)-DWI^23^, mitigates susceptibility artifacts, geometric distortion, and T2* blurring^24,25^ by dividing k-space acquisition into multiple segments, proving useful for assessing diffusion restrictions in optic nerve diseases^15^. The readout segmentation reduces the echo spacing and readout time, while the Nyquist sampling is retained for each spin excitation. Another approach, TGSE (turbo gradient spin echo)-BLADE-DWI^26–28^, employs radial k-space trajectories and k-space center oversampling to improve resistance to bulk motion artifacts, susceptibility artifacts and geometric distortion^29,30^. While these sequences seem promising for enhancing orbital imaging^6,15,24,31^, structured evaluations between rs-EPI-DWI, TGSE-BLADE-DWI and standard ss-EPI-DWI of ischemic retinal pathologies haven’t been reported yet. Previous studies and case reports have used rs-EPI-DWI as an alternative to ss-EPI-DWI for assessing retinal ischemia in acute vascular occlusions^11,32,33^. Boyko et al. reported higher interrater reliability for rs-EPI-DWI compared to ss-EPI-DWI^33^, whereas Harahsheh et al. found no significant difference^32^. Similarly, Lange et al. observed superior image quality of rs-EPI-DWI in detecting retinal vascular occlusions^11^. However, none of these studies included a quantitative comparison with TGSE-BLADE-DWI, and differences in MRI scanners, field strengths, and slice thicknesses may have influenced the results.

Our study aims to address this knowledge gap by systematically comparing the diagnostic performance of ss-EPI-DWI, rs-EPI-DWI, and TGSE-BLADE-DWI DWI - both quantitatively and qualitatively - using a customized DWI phantom simulating small ischemic lesions of various diameters embedded in healthy neuronal tissue. To the best of our knowledge, the present phantom study is the first to specifically examine sequence performance as a function of lesion size. Previous DWI phantom studies employing the same sequences have consistently relied on standardized phantoms containing either lesions of uniform size with varying Polyvinylpyrrolidone (PVP) concentrations^34–36^ or large homogeneous compartments^37^. Within these studies, TGSE-BLADE-DWI showed reduced ADC (apparent diffusion coefficient) bias and reduced geometric distortions compared to rs-EPI-DWI and ss-EPI-DWI^34,35^, as well as more homogeneous images using rs-EPI-DWI compared to ss-EPI-DWI^37^. However, although only the study by McDonald et al. evaluated all three sequences concurrently^34^, all investigations demonstrated marked differences in the acquisition times of the individual sequences. In order to emphasize the detection of very small lesions which is relevant with regard to the size of the retina, we systematically compare the diagnostic performance of ss-EPI-DWI, rs-EPI-DWI, and TGSE-BLADE-DWI depending on lesion size. At the same time, we placed emphasis on clinically comparable acquisition times for the individual sequences. Based on a phantom this study seeks to offer insights in and recommendations on an effective DWI sequence for diagnosing small acute ischemic retinal pathologies in ocular MRI.

## Methods

### Construction of a phantom

**Fig. 1:**
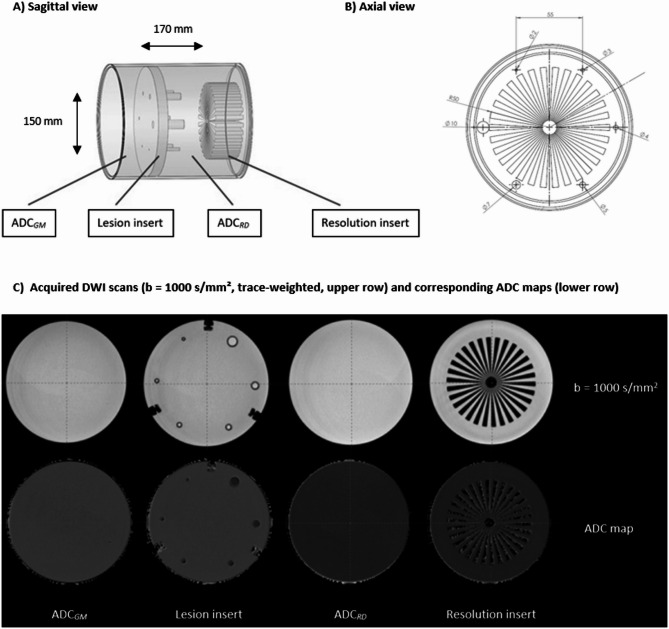
Schematic structure of the DWI phantom. The B-1000 trace-weighted images shown under (**C**) and the corresponding ADC map are examples from a scan series acquired using rs-EPI-DWI. The cylindrical phantom (height: 170 mm, diameter: 150 mm) comprises four compartments (from left to right, see **(**A) and (**C**)). The first compartment serves as a homogenous quality control region and simulates healthy neuronal tissue defined as gray matter with an ADC of 0.8 × 10⁻³ mm²/s (ADC_*GM*_). The second compartment contains six lesions simulating cytotoxic edema with restricted diffusion (ADC_*RD*_: 0.5 × 10⁻³ mm²/s) embedded in the ADC_*GM*_ matrix  (ADC_*GM*_: 0.8 × 10⁻³ mm²/s). The six lesions have diameters of 10 mm, 7 mm, 5 mm, 4 mm, 3 mm and 2 mm. The reduction in lesion size should serve to evaluate the sequences in the imaging of very small ischemic pathologies and at the same time make it possible to assess the influence of lesion size on image quality. The third compartment, also a quality control region, simulates a homogenous matrix of cytotoxic edema caused by restricted diffusion with an ADC of 0.5 × 10⁻³ mm²/s (ADC_*RD*_). Finally, a Siemens star at the phantom’s end facilitates resolution comparisons. Subpanels (**A**) and (**B**) source: Image © HQ Imaging, modified by the authors. Used with permission of Dr. Michael Bach.

Considering the mean retina thickness of ~ 280 μm, we simulated restricted diffusion (RD) on a larger scale by developing a custom-designed DWI MRI phantom in collaboration with HQ Imaging© (Heidelberg, Baden-Württemberg, Germany, Fig. 1). This phantom allowed for a comparative evaluation of the diagnostic performance of ss-EPI-DWI, rs-EPI-DWI and TGSE-BLADE-DWI.

To simulate ADC values in two compartments - healthy retinal tissue and ischemia-affected tissue - aqueous solutions with varying PVP concentrations were prepared within cylindrical tubes. Reference ADC values for healthy gray matter (0.8 × 10⁻³ mm²/s) and cytotoxic edema (0.5 × 10⁻³ mm²/s)^38–44^ guided these modifications. Specifically, an ADC of 0.5 × 10⁻³ mm²/s corresponded to a 42% PVP solution, and an ADC of 0.8 × 10⁻³ mm²/s to a 31% solution^45^. Because occlusion of the central retinal artery with subsequent ischemia mimics the pathophysiological features of a stroke with cytotoxic edema in the central nervous system, we regarded the corresponding ADC values within this phantom as appropriate. Since, in our view, there is currently no suitable phantom that can simulate retinal ischemia appropriately, we simulated the retina and adjacent eyeball as a circular lesion in our work (even though in vivo it is naturally only a thin layer “semicircular” in relation to the eyeball). In addition, we focused on imaging very small lesions, as this is one of the major challenges in imaging retinal ischemia. In order to analyze the diagnostic performance of diffusion-weighted sequences in relation to the size of the lesion, we considered it useful to use different lesion diameters. By progressively reducing the lesion size, we aimed to investigate trends in the diagnostic performance of the individual sequences to inform predictions and recommendations for their suitability in in-vivo applications.

To ensure accurate ADC measurements, the temperature dependency of diffusion during imaging was accounted for. The TGSE-BLADE-DWI transfers energy to tissue via rapid refocusing pulses^28^. As this energy is partially converted into heat and diffusion rates increase with temperature, the measured ADC values diverge from the modeled values based on a room temperature of 20 °C. To mitigate RF-induced heating, the phantom was equipped with an ethylene glycol based liquid glass thermometer for continuous temperature monitoring, which was located inside the phantom near the resolution insert (measuring range: 18–50 °C, graduations: 0.5 °C, accuracy: ± 1 °C). As all of the scans were conducted in succession (nearly three hours of scan time), the temperature of the phantom was read after each separate scan and the ADC corrected to the temperature at 20 °C by the formula^45^:

1 $$\:{\text{A}\text{D}\text{C}}_{20^\circ\:}=\:\frac{{\text{A}\text{D}\text{C}}_{measured}}{{\text{e}\text{x}\text{p}}^{\left(\text{c}\text{*}\left({\text{T}}_{\text{m}\text{e}\text{a}\text{s}\text{u}\text{r}\text{e}\text{d}}-20^\circ\:\text{C}\right)\right)}}$$

where ADC_*measured*_ is the ADC obtained from the ADC map, c is an individual correction factor (0.0315 for cytotoxic edema and 0.0297 for gray matter), and T_*measured*_ is the absolute temperature in °C.

### MRI protocol

Scans were conducted on a 3T MRI (Magnetom Vida, Siemens Healthineers©, Erlangen, Germany) using a 64-channel head-neck coil. Foam pads between the coil and phantom minimized motion induced by strong gradients (Fig. S1). Detailed imaging parameters are provided in Table 1. Our standard ss-EPI-DWI protocol has an acquisition time of 1.40 min. To achieve comparable scan durations among the sequences, b-1000-weighted ss-EPI-DWI images were averaged 12 times, increasing the total acquisition time to 5.09 min. Thus, the acquisition times of the sequences were within a range of 31 s (ss-EPI-DWI: 5.09 min, rs-EPI-DWI: 5.40 min, TGSE-BLADE-DWI: 5.28 min). Gradient non-linearity correction was conducted automatically by the vendor specific internal algorithm. Since the scanner’s interpolation algorithm for reducing the voxel size to 0.5 × 0.5 × 3 mm was available only for the ss-EPI-DWI and rs-EPI-DWI sequences, the TGSE-BLADE-DWI voxel size was interpolated post hoc using a MATLAB algorithm (R2024b, Version 24.2.0.2712019). Interpolation of TGSE-BLADE-DWI images was performed in the image domain using a bilinear interpolation model, which calculates each pixel value based on the four nearest neighboring pixels. We acknowledge that the interpolation algorithm applied to TGSE-BLADE-DWI may differ from vendor-specific implementations. However, as interpolation is enabled by default for ss-EPI-DWI and rs-EPI-DWI in the respective clinical sequence protocols, a corresponding adjustment was applied to TGSE-BLADE-DWI for consistency. Because increasing the default matrix for TGSE-BLADE-DWI (192 × 192 vs. 202 × 202 for the EPI sequences) would have resulted in a longer acquisition time, we aimed to achieve a comparable visual resolution within the same scan duration, even in the absence of the vendor-specific interpolation software. In this context, the slight reduction in the “true” spatial resolution of TGSE-BLADE-DWI was considered acceptable relative to the increase in acquisition time, as maintaining clinically feasible scan durations was prioritized. Further increases in the image matrix would have resulted in an additional loss of SNR per pixel for TGSE-BLADE-DWI. Additionally, achieving a more homogeneous signal pattern within the lesions was considered desirable, both to facilitate the determination of quantitative metrics and to improve visual assessment by the raters. Nonetheless, the original voxel size of 1.1 × 1.1 × 3 mm is reported for each sequence in the legend of Table 1.

**Table 1:** Imaging parameters of the conducted MRI scans.

Parameter	ss-EPI-DWI	rs-EPI-DWI (RESOLVE)	TGSE-BLADE-DWI	
MR acquisition type Slice thickness (mm) Number of slices FOV (mm) Matrix TR (ms) / TE (ms) b value (s/mm²) Diffusion preparation Voxel size (mm³) Number of averages Slice spacing Readout segments Echo spacing (ms) EPI echo train length Bandwidth (Hz/pixel) Phase oversampling Turbo factor Fip angle Diffusion mode Encoding direction BLADE coverage Acceleration mode	2D 3 20 220 × 220 202 × 202 5800 / 85 0,1000 Sjetskal Tanner 0.5 × 0.5 × 3.0* 2 (b0), 12 (b1000) 10% NA 0.96 76 1179 0% NA 90° (excitation) 4-scan trace A > > P NA GRAPPA	2D 3 20 220 × 220 202 × 202 10,270 / 60 0,1000 Sjektskal Tanner 0.5 × 0.5 × 3.0* 1 10% 7 0.34 11 (per segment) 688 0% NA 180° (refocusing) 4-scan trace A > > P NA GRAPPA	2D 3 20 220 × 220 192 × 192 4000 / 48 0,1000 Sjetskal Tanner 0.5 × 0.5 × 3.0* 1 10% NA 8.14 NA 840 100% 11 150° (refocusing) 4-scan trace A > > P 171.4% GRAPPA	
Acceleration factor	2	2	2	
Readout partial Fourier	NA	6/8	NA	
Phase partial Fourier	6/8	NA	NA	
Acquisition time	5:09 min.	5:40 min.	5:28 min.	

To match acquisition time, the b-1000-weighted ss-EPI-DWI images were averaged 12 times. As no scanner-based interpolation algorithm was available to adjust the TGSE-BLADE-DWI voxel size to that of the EPI sequences, post-hoc interpolation was performed using a MATLAB algorithm. *Original voxel size for all sequences was 1.1 x 1.1 x 3 mm. TR and TE were automatically adjusted by the device’s Syngo system based on factors such as the number of slices, signal averaging, and distance factor. Consequently, the TR was not manually set to its minimum value, which primarily affects the total acquisition time.

### Quantitative analysis

The three sequences were objectively evaluated by comparing ADC reproducibility, signal-to-noise-ratio (SNR), contrast-to-noise-ratio (CNR), relative contrast (ReCon), and geometric distortion rate (GDR) across the six lesion diameters. Image analysis was performed on b-1000 trace weighted scans using ITK-SNAP (v4.0.1, March 20, 2023) and MATLAB (R2024b, Version 24.2.0.2712019).

To first evaluate ADC reproducibility and accuracy in a homogeneous compartment, we defined a circular ROI of 74 mm³ in the center of the lesion insert for ADC_*GM*_ accuracy and in the compartment simulating RD for ADC_*RD*_ accuracy, thereby minimizing partial volume effects and interface artifacts. Subsequently, ROIs of 74 mm³, 36 mm³, 21 mm³, 12 mm³, 5 mm³ and 4 mm³ were placed in the insert corresponding to the lesion sizes with ITK SNAP. ADC values were adjusted to a constant temperature of 20 °C using the previously mentioned temperature-adapted ADC formula. For measuring the SNR and CNR, we used the same ROI areas as employed for the lesion specific ADC value analysis. The mean SNR was calculated by the following formula adapted from Dietrich et. al.^46^:

2 $$\:{\text{S}\text{N}\text{R}}_{mult.}=\:\frac{{\text{S}\text{I}}_{ROI(mult.)}}{{\text{S}\text{D}}_{ROI(mult.)}}$$

(SI_*ROI(mult.)*_ = mean signal intensity in the ROI of the 10 (multiple) b-1000-weighted scans, SD_*ROI(mult.)*_ = mean temporal standard deviation of the SI_ROI_ over the 10 (multiple) scans). Since multiple factors like phased array coils in combination with parallel imaging (as applied in our measurements), can introduce spatial signal non-uniformities^46^, the conventional “two-region” method - calculating the SNR as the ratio of mean signal intensity within the ROI to the SD of a background region - may yield inaccurate results. Therefore, we conducted a voxel-by-voxel analysis of the SD of the mean signal intensity within the ROI using ITK-SNAP. The time dependent stochastic variation of the SD was then averaged across all 10 repeated measurements per sequence and set into relation to each individual mean SI_*ROI*_. The ten SNR values were then averaged to the mean SNR_*mult.*_ mentioned in formula (2). For calculating the CNR, we modified formula (2) to:

3 $$\:{\text{C}\text{N}\text{R}}_{mult.}=\:\frac{{\text{S}\text{I}}_{ROI(mult.)}-{\text{S}\text{I}}_{GM(mult.)}}{{\text{S}\text{D}}_{GM(mult.)}}$$

(SI_*GM(mult.)*_ = mean signal intensity of the surrounding tissue over the 10 (multiple) b-1000-weighted scans, SD_*GM(mult.)*_ = mean temporal standard deviation of the SI_*GM*_ over the 10 (multiple) scans). The SI_*GM*_​ was determined as the mean signal intensity from four ROIs (≈ 170 mm³) placed adjacent to the lesions. A voxel-wise analysis of the SD of signal intensities within the four ROIs was also performed in this case, followed by CNR averaging across 10 scans (CNR_*mult.*_). To exclude noise variations and compare the contrast standardized, we also calculated the relative contrast (ReCon), using the formula^47^:

4 $$\:{\text{R}\text{e}\text{C}\text{o}\text{n}}_{ROI-GM}=\frac{{\text{S}\text{I}}_{ROI}-{\text{S}\text{I}}_{GM}}{{\text{S}\text{I}}_{ROI}+{\text{S}\text{I}}_{GM}}$$

To objectively assess the GDR, we analyzed the signal profile through the lesions. We created a mask and plotted the mean signal intensity of 360 signal profiles, offset by 1° from each other, across the lesions and the surrounding tissue using MATLAB software. In this analysis, the distance between the first two values at 10% above the two signal minima - which correspond to the edges of the lesion - were used to determine the lesion diameter. The measured diameter was then compared to the true diameter using the NEMA method^48^ and adapted the formula of Yokohama et al.^49^:

5 $$\:\text{G}\text{D}\text{R}=\frac{\left(\left|{\text{D}}_{DWI}-{\text{D}}_{t}\right|\right)}{{\text{D}}_{t}}*100\%$$

(D_*DWI*_ = measured diameter via signal profile, D_*t*_ = true diameter of the lesion).

### Qualitative analysis

For the qualitative evaluation, two neuroradiologists with over 10 years of experience independently rated the acquired b-1000 trace-weighted images and ADC maps based on six criteria using a five-point Likert scale from zero to four. The “HON” (Human Observer Network) image evaluation tool, proposed by Jahnke et al.^50^, was used for this purpose. The images were presented in a random order to ensure that the raters were blinded to the sequence type. They were allowed to adjust the image window, zoom in, and scroll through the image stack as needed. The raters were provided with a briefing document prior to the evaluation, which included essential information about the phantom’s compartment arrangement and the key structures to focus on during the assessment (Document S2). This preparatory material was intended to familiarize them with the phantom and ensure a consistent and informed evaluation process. The rating criteria included lesion visibility/contrast (0 = non-detectable, 1 = poor, 2 = moderate, 3 = good, 4 = excellent), susceptibility artifacts (0 = non-diagnostic/very severe artifacts, 1 = severe artifacts, 2 = moderate artifacts, 3 = mild artifacts, 4 = no artifacts), geometric distortion (0 = very severe distortion, 1 = considerable/severe, 2 = moderate distortion, 3 = mild distortion, 4 = no distortion), image noise (0 = very severe, 1 = severe, 2 = moderate, 3 = mild, 4 = no noise), resolution of the blades of the insert at the end of the phantom (0 = non-diagnostic, 1 = poor, 2 = moderate, 3 = good, 4 = excellent) and overall image quality (0 = non-diagnostic, 1 = poor, 2 = moderate, 3 = good, 4 = excellent). T2-weighted images were provided as references for evaluating geometric distortion. The mean ratings across different criteria were calculated and compared between the sequences.

### Statistical analysis

Each sequence was measured 10 times (*n* = 10 per sequence, *n* = 30 overall), and mean values of ADC, SNR, CNR, ReCon, and GDR were compared on the b-1000-weighted images using the Kruskal-Wallis test and pairwise Wilcoxon signed-rank test due to non-normal data distribution. We hypothesized that smaller lesion diameters might influence SNR, CNR, and GDR due to partial volume effects. Therefore, we assessed statistical correlations between these parameters and lesion diameter using the Spearman correlation coefficient, classifying correlation strengths as low (> 0.3), medium (> 0.5), and strong (> 0.7), also applying for negative values. For qualitative assessment, the raters evaluated the first five images per sequence (*n* = 15 overall) on the b-1000-weighted images, presented to them in a random order. The image series were selected without any specific criteria, as all images were acquired consecutively and thus the same examination conditions could be assumed. Weighted kappa statistics were calculated to determine interrater agreement, categorized according to Altman’s (1991)^51^ scale: poor (< 0.2), fair (0.21–0.4), moderate (0.41–0.6), good (0.61–0.8), and very good (0.81–1). Comparison of the mean rating criteria was analyzed by pairwise Wilcoxon-signed-rank test. Statistical analyses were conducted using R Studio (version 2025.05.1 + 513, “Mariposa Orchid”). For all statistical tests α was set to 0.05 and the tests were conducted two-sided.

## Results

### Quantitative results

#### Homogenous ADC_*GM*_ & ADC_*RD*_ accuracy

Over the course of approximately three hours of scanning, the temperature of the phantom initially measured 21 °C at the onset of the first ss-EPI-DWI acquisition. During the first ten scans, the temperature increased to 22 °C. A further rise of 0.5 °C was observed over the subsequent ten rs-EPI-DWI acquisitions, reaching 22.5 °C at the beginning of the final ten TGSE-BLADE-DWI scans. During these final scans, the phantom temperature remained stable at 22.5 °C.

**Fig. 2:**
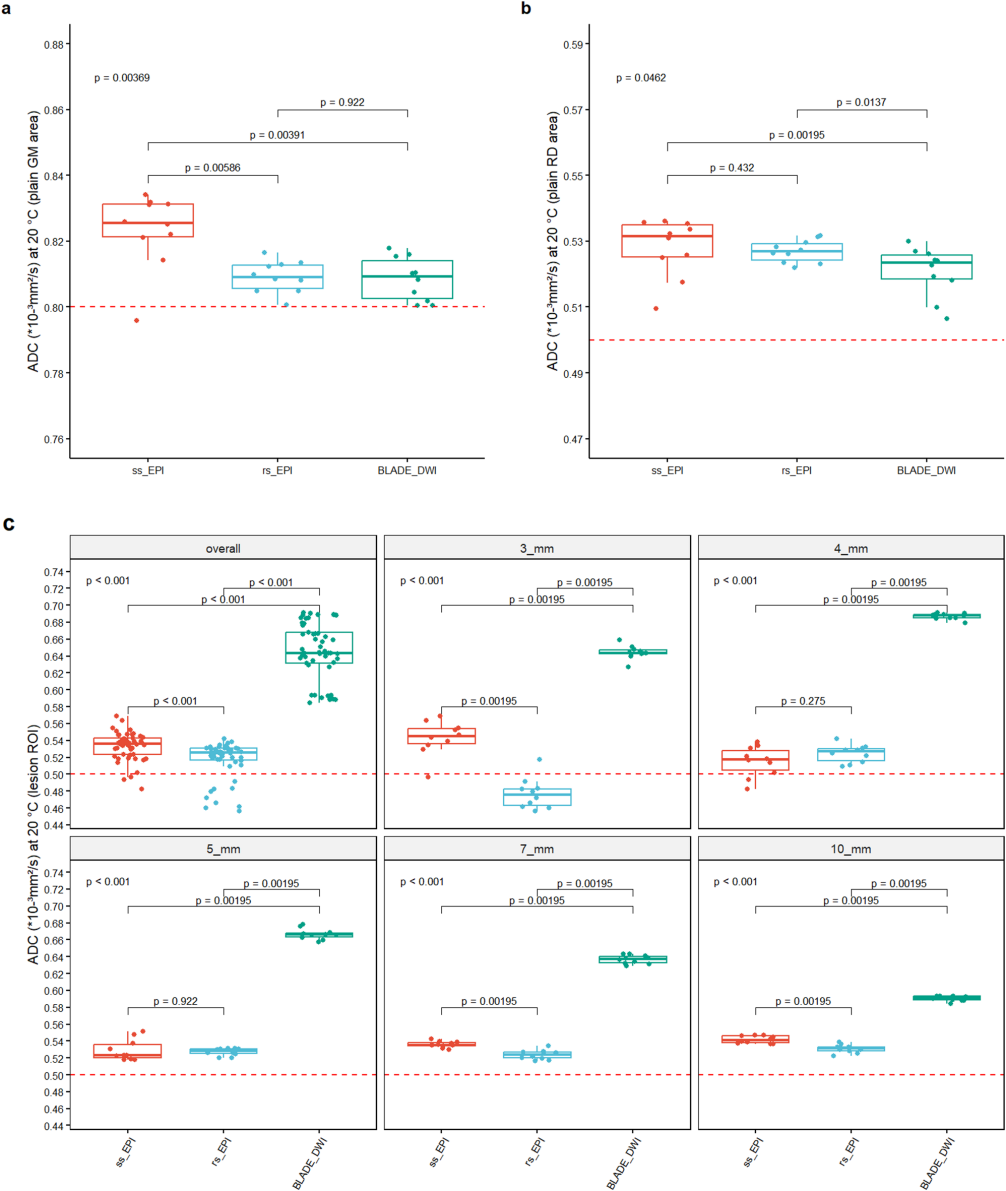
ADC accuracy comparison in homogenous areas and lesion specific. The reference ADC values for gray matter (0.8 × 10⁻³ mm²/s) and cytotoxic edema (0.5 × 10⁻³ mm²/s) are indicated by red dashed lines. (**a**) ADC_*GM*_ matrix: Both TGSE-BLADE-DWI and rs-EPI-DWI yield a mean ADC of (0.81 ± 0.01) × 10⁻³ mm²/s, demonstrating slightly higher accuracy compared to ss-EPI-DWI. (**b**) ADC_*RD*_ area: TGSE-BLADE-DWI achieves a more accurate mean ADC of (0.52 ± 0.01) × 10⁻³ mm²/s than the two EPI sequences. (**c**) Within each lesion ROI, rs-EPI-DWI achieves the highest ADC accuracy relative to the reference, whereas TGSE-BLADE-DWI exhibits the lowest accuracy. This discrepancy highlights that TGSE-BLADE-DWI measures less accurately in the peripheral lesion ROIs of the mixed compartment compared to the central 73 mm³ ROI in the homogeneous ADC_*RD*_ compartment. Meanwhile, ss-EPI-DWI fluctuates more around the reference value than rs-EPI-DWI, though it maintains similar precision for 4 mm and 5 mm lesions.

No significant ADC_*GM*_ differences were found between rs-EPI-DWI and TGSE-BLADE-DWI (rs-EPI-DWI: (0.81 ± 0.005) × 10⁻³ mm²/s, TGSE-BLADE-DWI: (0.81 ± 0.01) × 10⁻³ mm²/s, *p* = 0.92, Fig. 2a). However, ss-EPI-DWI showed the lowest accuracy ((0.82 ± 0.01) × 10⁻³ mm²/s) with substantial differences from TGSE-BLADE-DWI and rs-EPI (Fig. 2a). For ADC_*RD*_, TGSE-BLADE-DWI was closest to the reference ((0.52 ± 0.01) × 10⁻³ mm²/s) and differed considerably from ss-EPI-DWI and rs-EPI-DWI (Fig. 2b). Rs-EPI-DWI and ss-EPI-DWI yielded similar means ((0.53 ± 0.01) and (0.53 ± 0.003) × 10⁻³ mm²/s, respectively).

#### ADC accuracy of the lesion insert

When comparing the performance of the three sequences in the peripheral lesion ROIs, different ratios were observed. Due to the small size of the 2 mm lesion and voxel volume (0.5 × 0.5 × 3 mm), measurements were difficult to interpret and are omitted here for clarity. The same applies to SNR, CNR, ReCon, and GDR. Nevertheless, the 2 mm data are available in the supplementary information (Fig. S3, Table S4).

Across lesion sizes of 10 mm, 7 mm, 5 mm and 3 mm, rs-EPI-DWI yielded ADC values closest to the reference compared to ss-EPI-DWI and TGSE-BLADE-DWI (Fig. 2c; Table 2). Rs-EPI-DWI was more accurate than ss-EPI-DWI for 60% of lesions (10 mm, 7 mm, and 3 mm, Fig. 2c) and outperformed TGSE-BLADE-DWI at all sizes. TGSE-BLADE-DWI showed the largest deviation from the reference (Fig. 2c; Table 2). Averaging across all diameters, rs-EPI-DWI had the closest ADC to the reference (0.5 × 10⁻³ mm²/s), followed by ss-EPI-DWI and TGSE-BLADE-DWI. Given that ADC is calculated as − ln(S_*i*_/S_*0*_), the substantially higher absolute SI in the central ROI of the homogenous ADC_*RD*_ compartment for TGSE-BLADE-DWI b-1000-weighted images likely contrasts with that in small lesions at the insert. In contrast, the EPI-based sequences were less sensitive to lesion location, achieving more accurate ADC measurements.

#### Signal-to-noise-ratio (SNR)

**Fig. 3:**
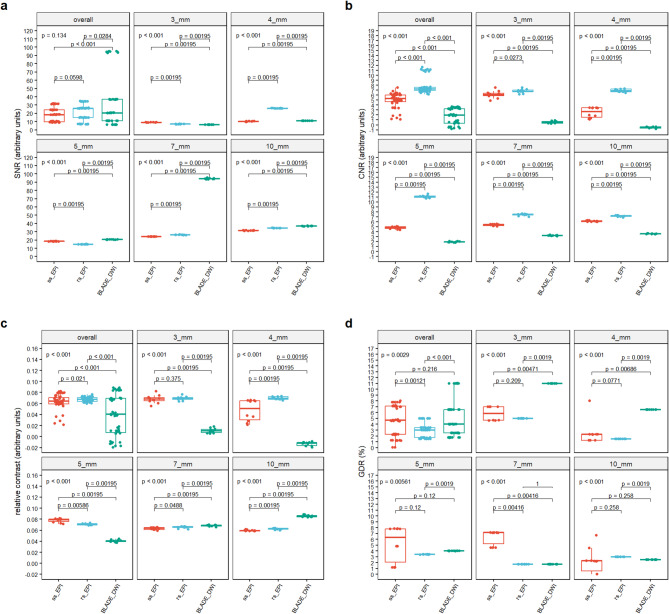
Comparison of SNR, CNR, ReCon and GDR. (**a**) Overall, no clear superiority between the two EPI sequences could be established in terms of SNR. On a per-lesion-size basis, rs-EPI-DWI outperformed ss-EPI-DWI in 60% of cases. TGSE-BLADE-DWI demonstrated higher SNR values than both EPI sequences in the larger lesions (10 mm, 7 mm, and 5 mm); however, the elevated values observed at 7 mm may be attributed to signal fluctuations rather than consistent performance. (**b**) With respect to SI_*GM*_ rs-EPI-DWI demonstrates clear superiority over the other sequences. In contrast to the SNR, TGSE-BLADE-DWI performs inferior to both EPI sequences. All sequences exhibit low variance in the measured values, indicating a homogeneous distribution of signal intensity and noise within the ROI. (**c**) When image noise is excluded, rs-EPI-DWI constantly outperforms ss-EPI-DWI, whereas TGSE-BLADE-DWI demonstrates a weaker contrast. (**d**) Both rs-EPI-DWI and TGSE-BLADE-DWI show minimal variability in GDR, indicating high precision. However, due to the overall poorer contrast in TGSE-BLADE-DWI, the D_*DWI*_ - derived from the signal curve through the mask - corresponds less accurately to the true value D_*t*_ compared to rs-EPI-DWI. Since the GDR values between rs-EPI-DWI and TGSE-BLADE-DWI were identical at 7 mm, no p value could be calculated for this comparison, which is the reason for the expression “1”.

Overall, no distinct difference in SNR performance was observed between the two EPI sequences (Fig. 3a; Table 2). On a lesion-specific level, rs-EPI-DWI exhibited higher SNR values for lesion sizes of 10 mm, 7 mm, and 4 mm, whereas ss-EPI-DWI demonstrated superior performance for lesions measuring 5 mm and 3 mm. TGSE-BLADE-DWI consistently outperformed both EPI sequences at lesion sizes of 10 mm, 7 mm, and 5 mm. However, at 4 mm, it showed inferior performance compared to rs-EPI-DWI, and at 3 mm, it yielded lower SNR values than both EPI sequences. The markedly elevated SNR values of TGSE-BLADE-DWI at 7 mm, relative to other lesion sizes and sequences, are likely attributable to random signal fluctuations. All sequences demonstrated relatively low measurement variability (Table 2), suggesting a stable distribution of signal and noise within the lesions, consistent with the static nature of the phantom model.

#### Contrast-to-noise-ratio (CNR)

**Fig. 4:**
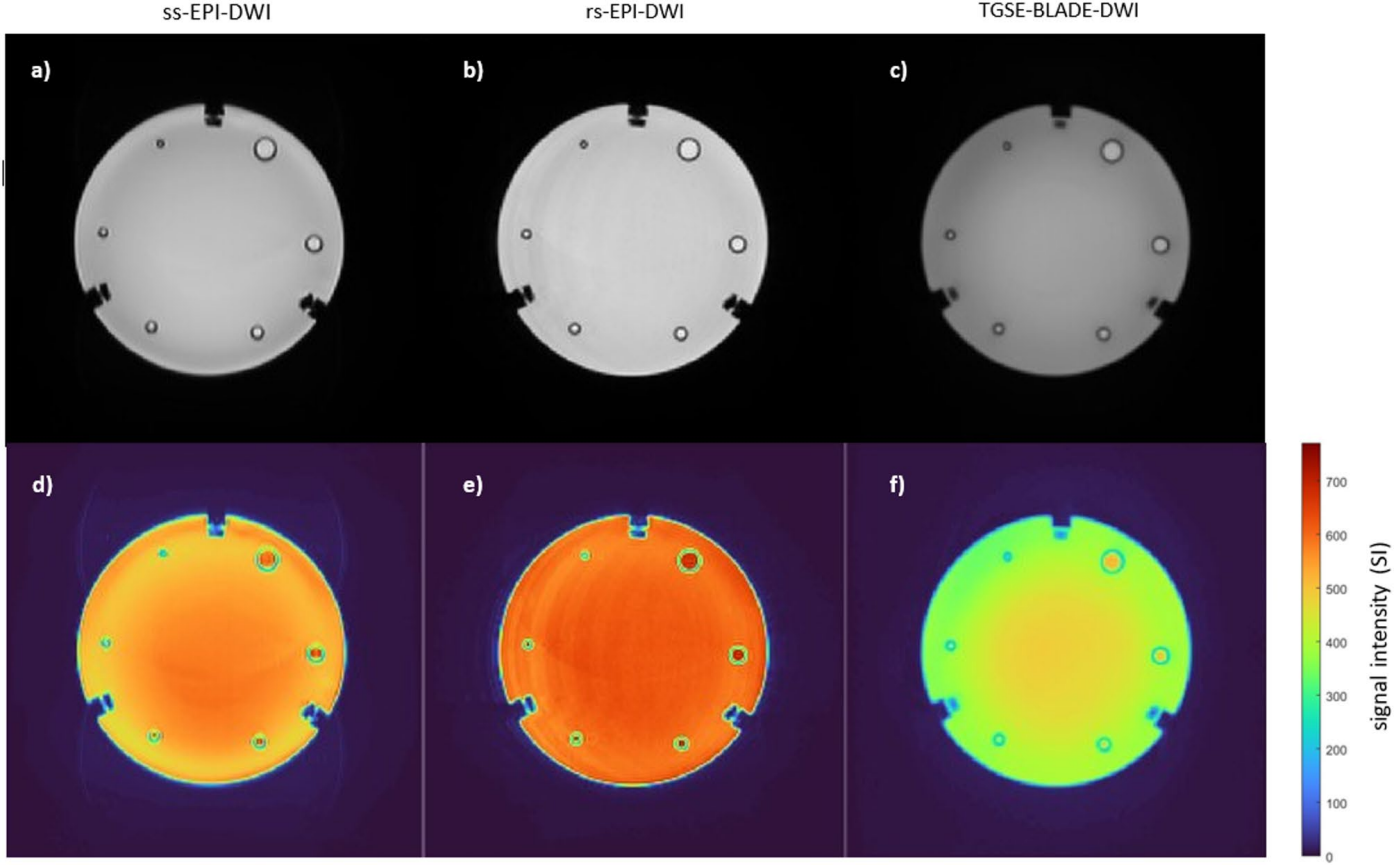
Averaged B-1000-weighted DICOMS of all 10 measurements per sequence merged with corresponding signal intensity scale. In all sequences, signal intensity (SI) increases from the phantom’s edge to its center. The highest SI is observed in rs-EPI-DWI (**b** + **e**), particularly within lesions, which explains its high ADC measurement accuracy. In the inter-lesion area, ss-EPI-DWI (**a ** + **d**) exhibits a less pronounced decrease in the SI on the heat map in relation to the lesion SI, accounting for its inferiority in CNR and relative contrast compared to rs-EPI-DWI. TGSE-BLADE-DWI (**c** + **f**) shows the lowest overall SI relation between lesion and inter-lesion area, corresponding to its poorer performance in quantitative metrics relative to the EPI sequences.

Analysis of the CNR across sequences demonstrates that rs-EPI-DWI consistently yields the highest CNR values, both for individual lesions and in aggregate (Fig. 3b; Table 2). In contrast, TGSE-BLADE-DWI exhibits the lowest CNR values, including negative values for lesions measuring 4 mm. This suggests an inversion of contrast at this lesion size, with lesion signal intensity falling below that of the surrounding tissue. The reason for the low CNR values of TGSE-BLADE-DWI relative to its SNR is illustrated in Fig. 4. While the mean signal intensity within the ROI is high relative to the temporal SD, resulting in a high SNR (mean SI ROI = 435.79, mean SD_*GM(mult.)*_ = 26.54), the mean signal intensity in the immediate vicinity of the ROI is approximately 426.49 with a temporal SD of 19.05. When applied to the corresponding CNR formula, this yields a relatively low value. Therefore, TGSE-BLADE-DWI provides accurate representation of local signals, but contrast with surrounding tissue remains limited. Across all sequences, CNR values show relatively low measurement variability, indicating a homogeneous distribution of signal and noise within and around the lesions, likely due to the calculation method based on CNR_*mult.*_ method​. A slight increase in measurement variance is observed only in ss-EPI-DWI at the 4 mm lesion size, possibly reflecting localized signal inhomogeneities in the perilesional region, which were not evident in the corresponding SNR analysis.

#### Relative contrast (ReCon)

When evaluating pure contrast - i.e., without accounting for temporally averaged noise within the ROI - the relative performance of the sequences shifts slightly (Fig. 3c; Table 2). TGSE-BLADE-DWI exhibits the highest relative contrast at a lesion size of 10 mm and marginally outperforms rs-EPI-DWI at 7 mm; however, this advantage appears to result primarily from a lower standard deviation in the measured values. Notably, TGSE-BLADE-DWI remains the only sequence to yield negative contrast values at 4 mm, reinforcing findings from the CNR analysis. The rs-EPI-DWI continues to outperform ss-EPI-DWI in nearly all lesion sizes, with the exception of 5 mm and 3 mm, and overall achieves the highest contrast values. This observation is further supported by Fig. 4, which highlights the elevated signal intensities associated with rs-EPI-DWI within the lesions and the pronounced SI decrease in the perilesional zone (mean SI_*ROI*_: 676.31 ± 3.9 at 10 mm, 686.63 ± 5.32 at 7 mm, 692.65 ± 6.78 at 5 mm, 687.77 ± 5.36 at 4 mm, 682.02 ± 7.03 at 3 mm; mean SI_*GM*_: 597.8 ± 4.24 at 10 mm, 602.58 ± 4.72 at 7 mm, 601.12 ± 5.63 at 5 mm, 598.0 ± 3.54 at 4 mm, 593.82 ± 2.97 at 3 mm).

#### Geometric distortion rate (GDR)

Rs-EPI-DWI demonstrated the best overall GDR performance among the sequences (Fig. 3d; Table 2). No remarkable differences were observed between ss-EPI-DWI and TGSE-BLADE-DWI overall. Notably, in contrast to the ss-EPI-DWI no scattering of the GDR values was observed in the rs-EPI-DWI and TGSE-BLADE-DWI acquisitions, which implies a very constant signal curve within the mask for calculating the GDR in these sequences. TGSE-BLADE-DWI slightly performed better than rs-EPI-DWI at 10 mm and better than ss-EPI-DWI at 7 mm. As GDR values of rs-EPI-DWI and TGSE-BLADE-DWI were identical at 7 mm, no p value could be calculated for this comparison, which is the reason for the expression “1” in the subpanel at Fig. 3d. However, TGSE-BLSADE-DWI performed worse than both sequences at 4 mm and 3 mm.

**Table 2:** Quantitative metrics of the three sequences.

	ss-EPI-DWI	rs-EPI-DWI (RESOLVE)	TGSE-BLADE-DWI
ADC_*20°*_	0.53 ± 0.02 (0.48–0.57) 0.54 ± 0.004 (0.54–0.55) 0.54 ± 0.004 (0.53–0.54) 0.53 ± 0.01 (0.52–0.55) 0.52 ± 0.02 (0.48–0.54) 0.54 ± 0.02 (0.5–0.57)	0.52 ± 0.02 (0.46–0.54) 0.53 ± 0.005 (0.52–0.54) 0.52 ± 0.01 (0.52–0.53) 0.53 ± 0.005 (0.52–0.53) 0.52 ± 0.01 (0.51–0.54) 0.48 ± 0.02 (0.46–0.52)	0.65 ± 0.03 (0.58–0.69) 0.59 ± 0.002 (0.58–0.59) 0.64 ± 0.004 (0.63–0.64) 0.67 ± 0.01 (0.66–0.68) 0.69 ± 0.004 (0.68–0.69) 0.64 ± 0.01 (0.63–0.66)
SNR	18.58 ± 8.55 (8.69–31.74) 31.42 ± 0.23 (31.14–31.74) 24.01 ± 0.06 (23.92–24.12) 18.32 ± 0.2 (18.0–18.52) 10.16 ± 0.44 (9.61–10.62) 8.97 ± 0.18 (8.69–9.23)	21.65 ± 9.78 (6.76–34.81) 34.41 ± 0.12 (34.19–34.81) 26.19 ± 0.2 (25.89–26.54) 14.78 ± 0.14 (14.63–15.01) 26.02 ± 0.2 (25.71–26.34) 6.87 ± 0.07 (6.76–6.96)	33.74 ± 32.33 (6.07–95.24) 36.82 ± 0.18 (36.62–37.16) 94.21 ± 0.75 (93.15–95.24) 20.46 ± 0.14 (20.18–20.59) 11.06 ± 0.04 (10.99–11.11) 6.12 ± 0.04 (6.07–6.23)
CNR	4.98 ± 1.48 (1.07–7.49) 6.12 ± 0.11 (5.99–6.31) 5.39 ± 0.17 (5.09–5.61) 4.82 ± 0.25 (4.38–5.09) 2.46 ± 1.05 (1.07–3.48) 6.12 ± 0.73 (4.87–7.49)	7.87 ± 1.66 (6.19–11.65) 7.15 ± 0.15 (6.87–7.32) 7.43 ± 0.21 (6.99–7.65) 11.09 ± 0.26 (10.69–11.65) 6.91 ± 0.31 (6.43–7.27) 6.79 ± 0.4 (6.19–7.53)	1.76 ± 1.59 (-0.8–3.67) 3.58 ± 0.05 (3.52–3.67) 3.24 ± 0.06 (3.12–3.35) 1.95 ± 0.1 (1.82–2.13) -0.53 ± 0.15 (-0.8 - -0.35) 0.53 ± 0.22 (0.25–0.89)
ReCon	0.06 ± 0.01 (0.02–0.08) 0.06 ± 0.001 (0.06–0.06) 0.06 ± 0.002 (0.06–0.07) 0.08 ± 0.004 (0.07–0.08) 0.05 ± 0.02 (0.02–0.07) 0.07 ± 0.01 (0.06–0.08)	0.07 ± 0.004 (0.06–0.08) 0.06 ± 0.001 (0.06–0.06) 0.07 ± 0.002 (0.06–0.07) 0.07 ± 0.001 (0.07–0.07) 0.07 ± 0.003 (0.07–0.07) 0.07 ± 0.004 (0.06–0.08)	0.04 ± 0.04 (-0.02–0.09) 0.09 ± 0.002 (0.08–0.09) 0.07 ± 0.001 (0.07–0.07) 0.04 ± 0.002 (0.04–0.04) -0.01 ± 0.003 (-0.02 - -0.01) 0.01 ± 0.004 (0.01–0.02)
GDR	4.42 ± 2.62 (0–8) 2.26 ± 2.11 (0–6.7) 6.37 ± 1.24 (4.57–7.14) 5.22 ± 3.02 (1.2–7.8) 2.43 ± 2.02 (1.25–8) 5.83 ± 1.23 (4.67–7)	2.92 ± 1.28 (1.5–5) 3 ± 0 (3–3) 1.71 ± 0 (1.71–1.71) 3.4 ± 0 (3.4–3.4) 1.5 ± 0 (1.5–1.5) 5 ± 0 (5–5)	5.14 ± 3.39 (1.71–11) 2.5 ± 0 (2.5–2.5) 1.71 ± 0 (1.71–1.71) 4 ± 0 (4–4) 6.5 ± 0 (6.5–6.5) 11 ± 0 (11–11)

Values are displayed as mean ± SD (Min./Max.) and arranged in order of lesion size from top to bottom: total value, 10 mm, 7 mm, 5 mm, 4 mm, 3 mm.

#### Effect of lesion size on ADC value, SNR, CNR, Recon and geometric distortion

**Fig. 5:**
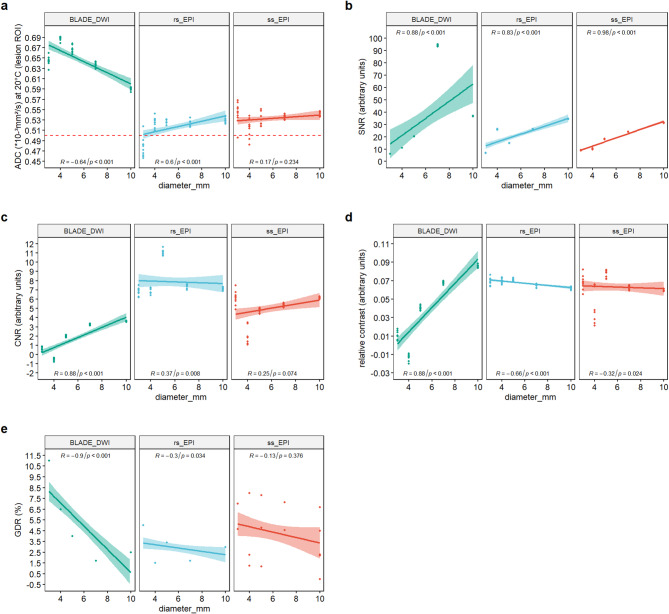
Correlation between the evaluated metrics and lesion size. (**a**) The reference ADC value (0.5 × 10⁻³ mm²/s) is shown as a red dashed line. Even though ADC values from the EPI sequences deviate more from the reference value with increasing lesion size, they show enhanced ADC accuracy compared to the TGSE-BLADE-DWI across all lesion sizes. (**b**) All sequences exhibit a strong positive correlation between SNR and lesion size, with the most pronounced effect observed in the ss-EPI-DWI sequence. (**c**) Incorporating SI_*GM*_ in the CNR calculation constantly yields a strong correlation with lesion size for TGSE-BLADE-DWI. The two EPI sequences exhibit a markedly lower correlation between CNR and lesion size, suggesting relatively consistent performance across the full range of lesion sizes. (**d**) Although EPI sequences exhibit marginally better contrast for smaller lesions, TGSE-BLADE-DWI shows a positive correlation between ReCon and lesion size, indicating enhanced contrast for larger lesions. (**e**) All sequences demonstrate reduced geometric distortion with increasing lesion size, with considerable reductions observed only for rs-EPI-DWI and TGSE-BLADE-DWI.

Figure 5a shows that temperature-corrected ADC values from TGSE-BLADE-DWI approach the true value of 0.5 × 10⁻³ mm²/s as lesion size increases, whereas the two EPI sequences appear to be slightly more accurate in the range of smaller lesions. Nevertheless, the ADC accuracy of the two EPI sequences is closer to the specified value of 0.5 × 10⁻³ mm²/s compared to TGSE-BLADE-DWI throughout all lesion sizes. TGSE-BLADE-DWI demonstrates a slightly stronger correlation between lesion size and ADC values compared to rs-EPI-DWI, both with medium-strength correlations according to Cohen’s criteria. For ss-EPI-DWI, no substantial correlation is observed between lesion diameter and ADC values. Across all sequences, SNR values were notably higher in regions containing larger lesions, indicating a strong positive correlation between lesion size and SNR (Fig. 5b). This correlation was observed in all sequences but appeared most pronounced in the ss-EPI-DWI sequence. A similar positive correlation between CNR and lesion size is observed for TGSE-BLADE-DWI as for the SNR (Fig. 5c). While both rs-EPI-DWI and ss-EPI-DWI also show a positive correlation between CNR and lesion size, the strength of this relationship is markedly lower than that observed for TGSE-BLADE-DWI. This suggests that the two EPI-based sequences exhibit more consistent performance across varying lesion sizes. When evaluating relative contrast without incorporating the temporally averaged noise, an inverse correlation with lesion size is observed for both EPI sequences (Fig. 5d), which is even more pronounced for the rs-EPI-DWI. This indicates improved contrast for smaller lesions in rs-EPI-DWI and ss-EPI-DWI. In contrast, TGSE-BLADE-DWI continues to demonstrate a strong positive correlation, suggesting improved lesion visualization within surrounding tissue as lesion size increases. Finally, an inverse correlation is observed for all sequences with respect to GDR, where smaller lesion diameters are associated with greater geometric distortion (Fig. 5e). TGSE-BLADE-DWI again exhibits the strongest correlation with lesion size, suggesting that the measurement of the GDR based on signal mask calculation is substantially more accurate in the presence of larger lesions. For the EPI sequences, this effect is weaker, with only the GDR of rs-EPI-DWI showing a substantial correlation with lesion diameter (Fig. 5e). As shown in Fig. 3d, ss-EPI-DWI demonstrates the greatest variability in measured values. Although the GDR is consistently higher than that of rs-EPI-DWI, the lack of a substantial correlation with lesion size is likely attributable to the greater dispersion of the measurements. Overall, TGSE-BLADE-DWI demonstrates the strongest correlation between lesion size and quantitative imaging metrics, whereas the EPI-based sequences exhibit a comparatively weaker dependence.

### Qualitative results

No differences were observed in contrast / lesion visibility (Table 3). Rater one consistently rated contrast as “good” across all sequences, whereas rater two rated one ss-EPI-DWI image as “moderate.” Due to minimal rating variance, Ƙ approached zero. However, the raters agreed on 14 of 15 images (93% agreement), though potential random effects were not considered.

Differences were found regarding susceptibility artifacts. Both raters observed most artifacts in ss-EPI-DWI. For rater two, differences were observed in both comparisons, whereas for rater one, only TGSE-BLADE-DWI differed substantially from ss-EPI-DWI. Ƙ indicated moderate agreement (0.57). The raters provided identical assessments in 10 of 15 cases, yielding approximately 67% agreement.

Geometric distortion in phantom structures was more pronounced in the ss-EPI-DWI than in the other two sequences, as assessed by both raters. Ƙ was moderate (0.42), with 60% agreement (9 of 15 identical ratings). Rater two classified four ss-EPI-DWI images as “mild” and the remainder as “moderate,” whereas rater one rated three ss-EPI-DWI images as “moderate” and two as “severe,” two rs-EPI-DWI images as “moderate” and three as “mild,” and one TGSE-BLADE-DWI image as “no distortion”, with the rest rated as “mild.”

For rater one, TGSE-BLADE-DWI exhibited the lowest image noise, while rater two favored rs-EPI-DWI, with significance reached only for rater one (*p* = 0.02). Despite 73% agreement (11 of 15 identical ratings), Ƙ was moderate at 0.41. Rater one classified four ss-EPI-DWI and two rs-EPI-DWI images as “moderate,” with all others rated “mild.” Rater two deemed three ss-EPI-DWI and one TGSE-BLADE-DWI recordings as “moderate,” with the remainder rated “mild.”

The resolution quality of radial structures in the resolution insert followed a pattern similar to general contrast. No remarkable differences were found in mean ratings among the three sequences. Variability was minimal, as reflected in the low Ƙ. Rater one rated 14 of 15 images as “good,” assigning a “moderate” rating to one ss-EPI-DWI image. Rater two rated 11 images as “good” and four as “moderate,” penalizing two ss-EPI-DWI and two TGSE-BLADE-DWI images. The raters agreed on 10 of 15 cases, yielding ≈ 67% agreement.

The overall image quality assessment by both raters indicates that the ss-EPI-DWI sequence performed worst. However, substantial differences were only observed for rater two, specifically between the rs-EPI-DWI and ss-EPI-DWI sequences. While rs-EPI-DWI was rated higher than TGSE-BLADE-DWI, the difference was not statistically significant (*p* = 0.42). For rater one, TGSE-BLADE-DWI appeared to perform best, though no significant superiority was demonstrated (*p* = 0.067, *p* = 0.42). Interrater agreement, with a Ƙ of 0.53, was moderate despite high rating homogeneity. Rater one rated four images as “moderate” (three ss-EPI-DWI, one rs-EPI-DWI), with all others rated as “good.” Rater two rated three ss-EPI-DWI, one rs-EPI-DWI, and one TGSE-BLADE-DWI image as “moderate,” with the rest rated as “good.” The raters agreed on 12 of 15 images, yielding 80% agreement.

**Table 3:** Results of the qualitative evaluation by two neuroradiologists.

Rating criteria	Rater 1				Rater 2				Ƙ (95% CI)
	ss-EPI	rs-EPI	TGSE-BLADE	*p*-value	ss-EPI	rs-EPI	TGSE-BLADE	*p*-value	
Contrast	3 ± 0	3 ± 0	3 ± 0	1*^,^**^,^***	2.8 ± 0.4	3 ± 0	3 ± 0	0.42*^,^ ** NA***	0 (0–0)
Susceptibility artifacts	2 ± 0	2.4 ± 0.5	3.2 ± 0.4	0.18* 0.01** 0.06***	2 ± 0	3 ± 0	3 ± 0	0.004*^,^** NA***	0.57 (0.26–0.88)
Geometric distortion	1.6 ± 0.5	2.6 ± 0.5	3.2 ± 0.4	0.04* 0.009** 0.12***	2.2 ± 0.4	3 ± 0	3 ± 0	0.02*^,^ ** NA***	0.42 (0.05–0.78)
Image noise	2.2 ± 0.4	2.6 ± 0.5	3 ± 0	0.27* 0.02** 0.18***	2.4 ± 0.5	3 ± 0	2.8 ± 0.4	0.067* 0.27** 0.42***	0.41 (-0.05-0.87)
Resolution	2.8 ± 0.4	3 ± 0	3 ± 0	0.42*^,^ ** 1***	2.6 ± 0.5	3 ± 0	2.6 ± 0.5	0.18*^,^ *** 1.0**	-0.1 (-0.32-0.08)
Overall image quality	2.4 ± 0.5	2.8 ± 0.4	3 ± 0	0.27* 0.067** 0.42***	2.2 ± 0.4	3 ± 0	2.8 ± 0.4	0.02*, 0.09** 0.42***	0.53 (0.06–0.99)

All sequences resolved even the smallest lesions with good quality, with no significant differences between raters regarding contrast or resolution. Notably, rs-EPI-DWI and TGSE-BLADE-DWI significantly outperformed ss-EPI-DWI in terms of susceptibility artifacts and geometric distortion, while overall image quality remained largely unaffected. The Ƙ values primarily reflect the raters’ consistent rating behavior, resulting in minimal variability. Consequently, percentage agreement for each quality level was calculated, despite its inability to account for potential random effects (see section Qualitative Results) *= ss-EPI-DWI vs. rs-EPI-DWI, **= ss-EPI-DWI vs. TGSE-BLADE-DWI, ***=rs-EPI-DWI vs. TGSE-BLADE-DWI, values displayed as mean ± SD, 1 = p value is not defined as all differences are zero, α ≤ 0.05 was considered to be statistically significant.

## Discussion

This phantom study systematically compared ss-EPI-DWI, rs-EPI-DWI, and TGSE-BLADE-DWI in visualizing small foci of ischemia-induced cytotoxic edema. The results aim to identify the most suitable sequence for lesions from 10 mm down to 2 mm in size and to guide imaging of acute ischemic retinal pathologies.

Consistent with previous literature^15,18,29,52^, our phantom study confirmed that rs-EPI-DWI and TGSE-BLADE-DWI considerably reduce susceptibility artifacts and geometric distortions compared to ss-EPI-DWI. Local magnetic field fluctuations at air-tissue and tissue-bone interfaces cause discrepancies between the intended gradient field and the actual magnetic field. In ss-EPI-DWI, the weak phase-encoding gradient heightens sensitivity to these inhomogeneities, resulting in voxel misalignment^53^ in phase encoding direction. In contrast, rs-EPI-DWI benefits from shorter echo spacing for faster k-space traversal in phase encoding direction, while TGSE-BLADE-DWI employs multiple echo trains and a phase-insensitive preparation module to eliminate non-CPMG (Carr-Purcell-Meibom-Gill) components and therefore minimize phase inconsistencies and b0-related artifacts^28,54^. In our phantom susceptibility artifacts were primarily observed at the edges of the cylinder and at the matrix-lesion interface. Furthermore, ss-EPI-DWI images exhibited more T2* blurring than rs-EPI-DWI, although lesion resolution was not qualitatively affected. This increased blurring arises from ss-EPI’s single-excitation acquisition of k-space, which causes greater T2* dephasing and peripheral signal loss, therefore especially degrading spatial information^53^. In contrast, the TSE-based TGSE-BLADE-DWI is largely unaffected by T2* decay.

There is ongoing debate regarding the SNR and CNR performance of ss-EPI-DWI, rs-EPI-DWI, and TGSE-BLADE-DWI, especially between ss-EPI-DWI and rs-EPI-DWI. Kida et al. and Fries et al. reported lower SNR for TGSE-BLADE-DWI compared to ss-EPI-DWI - with Fries et al. also noting lower CNR - using a 32-channel head coil and parameters similar to our study^55,56^. In contrast, Fu et al. found no remarkable CNR differences among the sequences in cerebellopontine angle tumors, although rs-EPI-DWI showed substantially higher SNR than ss-EPI-DWI^29^. Conversely, Xu et al. reported lower SNR for rs-EPI-DWI while also no considerable CNR differences emerged in the imaging of orbital tumors^24^; however, they observed superior contrast in rs-EPI-DWI when comparing absolute signal intensities. With regard to the phantom studies, TGSE-BLADE-DWI showed an SNR value almost identical to ss-EPI-DWI^34^, or a lower value depending on the number of excitations and the selected slice thickness^35^. Xu et al. and Kim et al. concluded that SNR and CNR depend on multiple factors such as TE, the number of signal averages, and tissue T2 relaxation times^24,57^. Next to these factors, it should be noted that the method of SNR and CNR calculation, as well as the application of parallel imaging techniques, can significantly influence the results. For instance, Kida et al., Fries et al. as well as McDonald et al. and Ohki et al. employed the subtraction method to estimate image noise^34,35,55,56^, whereas Xu et al. and Fu et al. utilized the conventional “two-region” approach based on SI_*ROI*_ / SD_*BG*_^24,29^. However, the use of parallel imaging and various multi-channel phased array coils results in a noise distribution that is neither statistically nor spatially homogeneous^46^. Consequently, SNR and CNR calculations based on the SI_*ROI*_ / SD_*BG*_ method may be inaccurate and can lead to distorted results. This variability underscores the importance of the selected calculation method. In our study, the use of a static phantom combined with multiple acquisitions per sequence allowed for the calculation of SNR and CNR based on the averaged temporal SD within a defined ROI. This approach was considered appropriate, as it provided a more reliable estimation under the assumption of a stochastically homogeneous noise distribution.

With regard to lesion size dependency all evaluated sequences qualitatively resolved lesions as small as 2 mm with sufficient clinical clarity; however, rs-EPI-DWI provided the most reliable quantitative metrics, including ADC values, even for the smallest lesions. Bogner et al. demonstrated that combining rs-EPI-DWI with GRAPPA at 7T yields high-resolution imaging with a submillimeter detection rate in breast tumors^58^. Similarly, with regard to retinal imaging, studies by Danyel et al. and Siebert et al. indicate that higher field strengths (3T vs. 1.5T) improve detection of retinal diffusion restrictions in the setting of acute central retinal artery occlusion, although mild cytotoxic edema in the inner retinal layer remains challenging^10,12^. These findings suggest that applying GRAPPA with optimized acceleration factors at higher field strengths could further enhance the utility of rs-EPI-DWI for detecting small ischemic retinal pathologies. A particularly strong relationship was observed between lesion size and quantitative metrics in TGSE-BLADE-DWI. This may be partially attributed to the sequence-specific point spread function (PSF), which characterizes the spatial distribution of signal intensity. According to Pipe and Zwart, in the context of the turboprop technique, off-resonance effects do not shift the PSF or alter the width of its main lobe^28^ - phenomena that typically manifest as image blurring in EPI-based sequences. However, off-resonance effects do reduce local signal amplitude. This reduction is likely due to the blade-based acquisition scheme, in which overlapping data before and after the spin echo can interfere destructively with off-resonant spins. These effects are particularly pronounced in the central region of k-space, where they may impact the fidelity of signal reconstruction^28^. The signal loss is particularly evident in smaller lesions, where the ROI comprises only a few voxels. This may be an explanation why TGSE-BLADE-DWI yielded very accurate ADC values in homogeneous compartments, while a reduction in accuracy was observed lesion-specific. Moreover, variations in tissue composition - specifically their T2 and T2* relaxation times - exert distinct effects on the PSF. In the phantom used for this study, T2 values of 730 ms and 544 ms have been reported for PVP concentrations of 31% and 42%, respectively^59^. Although no specific reference values are available for the corresponding T2* times, they are expected to be shorter. The T2 relaxation time predominantly influences signal amplitude and is therefore especially relevant for TGSE-BLADE-DWI. In contrast, T2* decay leads to an exponential temporal signal attenuation along the phase-encoding direction, predominantly affecting EPI-based sequences. EPI-based techniques must minimize the influence of T2* decay on the PSF by rapidly acquiring k-space data along the phase-encoding direction with e.g. the use of shorter echo spacing or employing k-space segmentation. Optimization of TGSE-BLADE-DWI acquisition parameters appears necessary to enhance local signal intensity, thereby improving the detection of small ischemic lesions. A slight increase in TR and a reduction in TE may boost SNR. Adjusting the ETL and turbo factor is necessary to balance scan efficiency and signal strength^28^, and reducing receiver bandwidth could help minimize noise. Although increasing voxel size might capture more signal, this may compromise the resolution needed for depicting microstructural retinal ischemia. Additionally, optimizing b-values could enhance diffusion contrast. However, as this study employed the clinic’s internal sequence parameters within the stroke protocol, the comparison of the sequences was primarily based on acquisition time and the image resolution achievable within this constraint (see methods section).

The influence of the PSF also has an impact on the partial volume effect (PVE). PVE arises when different tissues with distinct signal intensities are averaged within a single voxel, resulting in a composite intensity value. In our phantom study, PVE was predominantly observed at the lesion-matrix interface, with its impact increasing as lesion size decreased. Although PVE had little qualitative effect on lesion visibility, it substantially altered signal intensity and contrast in quantitative analyses. Especially for the TGSE-BLADE-DWI the influence of the PSF on PVE impacted the calculation of the GDR. Due to the averaging of signal intensities within small regions, it became increasingly difficult to accurately determine the distance between the two points above the signal minima, thereby limiting the reliability of GDR measurements. Consequently, when interpreting the correlation between lesion size and quantitative metrics, the amplitude of the PSF and its effect on PVE should be carefully considered - especially in the context of TGSE-BLADE-DWI. PVE may therefore possibly impact retinal imaging due to the retina’s composition of 10 distinct histological layers and its location between the vitreous body and the choroidal layer. This anatomical complexity causes multiple tissue types to converge within a limited spatial area, leading to pronounced PVE at high image resolutions. In 2000, Ballester et al. introduced a mixed-model approach that utilizes statistical models to estimate the probability distribution of tissue fractions within voxels, thereby enhancing sub-voxel accuracy^60^. Although various strategies - particularly in diffusion tensor imaging to reduce CSF contamination^61,62^ - have been developed to mitigate PVE, some of these methods compromise SNR^62^, limiting the detection of very small lesions. Thus, further methodological advancements are necessary to address PVE while preserving signal fidelity for precise visualization of ischemic retinal lesions.

Next to the implementation of optimized sequence parameters and image post-processing the selection of an appropriate coil system is crucial for enhanced local signal acquisition. In our study, we employed a 64-channel head-neck coil. Erb-Eigner et al.^63^ demonstrated in a phantom study at 1.5T that a 6 cm loop surface coil is optimal for orbital imaging, while combining multiple surface coils or adding a head coil increased noise and reduced SNR. In a subsequent study, the same group^64^ suggested that coil choice has a greater impact on image quality than MRI field strength, as SNR improves with decreasing coil size and closer proximity to the target structure. Future research should evaluate how the integration of ss-EPI-DWI, rs-EPI-DWI, or TGSE-BLADE-DWI with loop surface coils influences orbital imaging quality both quantitatively and qualitatively.

Our study also has limitations. Firstly, we used a phantom model rather than a patient-based analysis, which does not account for the human eye’s and orbit’s individual tissue composition—resulting in variations in T1 and T2 relaxation times, SNR, and CNR. Additionally, the diffusion properties of protons in an aqueous PVP solution differ from those in retinal nerve cell layers, potentially leading to discrepancies in detecting diffusion restrictions. Given the availability of only retrospective conventional ss-EPI-DWI and rs-EPI-DWI data for CRAO^9–12^, we adopted a phantom-based approach as an initial step, enabling a systematic evaluation of the three sequences for detecting small ischemic lesions. The insights from this study will form the basis for future investigations with volunteers and patients. Moreover, while TGSE-BLADE-DWI offers improved motion artifact correction, its benefits could not be fully exploited in a static phantom. In CRAO, sudden loss of visual acuity may cause patient restlessness, increasing motion during examinations; hence, future patient studies using TGSE-BLADE-DWI are expected to demonstrate a substantial reduction in motion artifacts, as indicated by previous research^26,31,47,65^. Finally, various sequence parameters, including TR/TE, number of averages, b-values, matrix size, base resolution, etc. influence image contrast and signal intensity. In this study, clinical internal sequence protocols for the detection of ischemic CNS lesions were employed, with the aim of achieving comparability across sequences, particularly in the trade-off between acquisition time and resolution. As described in the methods section, this required the application of an external interpolation algorithm for TGSE-BLADE-DWI, which may differ from the vendor-specific implementation. Nevertheless, this approach was considered appropriate for enabling comparison of the three sequences without extending acquisition time, particularly in the evaluation of very small lesions. Further studies are warranted to investigate the impact of factors such as TR/TE, b-values, voxel size, and potential interpolation algorithms on the depiction of small ischemic lesions. Our study may serve as a reference for future in vivo and in vitro comparative analyses.

In summary, rs-EPI-DWI and TGSE-BLADE-DWI appear to be a promising complement to conventional ss-EPI-DWI in microstructural orbital imaging. However, an individualized assessment is essential to select the most suitable sequence for specific clinical applications. If the primary goal is to achieve higher local SNR and CNR alongside precise ADC values, despite residual susceptibility and motion artifacts, rs-EPI-DWI is preferable. Conversely, TGSE-BLADE-DWI may be advantageous when susceptibility artifacts remain problematic or when patient motion is a significant concern. Future studies should aim to integrate the benefits of both sequences by addressing PVE and optimizing acquisition times to enhance clinical feasibility, as very small lesions still pose challenges for TGSE-BLADE-DWI in particular. Strategies may include refining sequence parameters, employing higher field strengths and specialized coils, incorporating parallel imaging with appropriate acceleration factors, and utilizing advanced post-processing techniques to further improve image quality and diagnostic accuracy. To validate the transferability of our findings to the human organism and to assess the clinical applicability of the proposed sequences, further comparative analyses of rs-EPI-DWI and TGSE-BLADE-DWI with conventional ss-EPI-DWI are required, initially in test subjects and subsequently in patients. This approach enables a more comprehensive investigation of the specific sequence requirements for the orbital and periocular region, thereby facilitating an evaluation of the clinical utility of ss-EPI-DWI, rs-EPI-DWI and TGSE-BLADE-DWI in routine microstructural orbital MRI.

## Supplementary Information

Below is the link to the electronic supplementary material.

Supplementary Material 1

## Data Availability

The data that support the findings of this study are available from the corresponding author upon reasonable request.
