# Supplementary material for: The diagnostic performance of ss-EPI-DWI, rs-EPI-DWI (RESOLVE) and TGSE-BLADE-DWI in a model of retinal ischemia: a comparative phantom study
Source: Sci Rep. 2025 Dec 3;15:43112. doi: 10.1038/s41598-025-30769-w (PMC12678797; doi:10.1038/s41598-025-30769-w)
Supplement: Supplementary file 1 — Supplementary Material 1 [file 41598_2025_30769_MOESM1_ESM.pdf]

Supporting Information

Figure S1

Setup of the phantom in the 64-channel head coil. *The spaces between the phantom and the coil were padded with foam to prevent the phantom from moving during the measurements. With permission from Yannick Schröder*

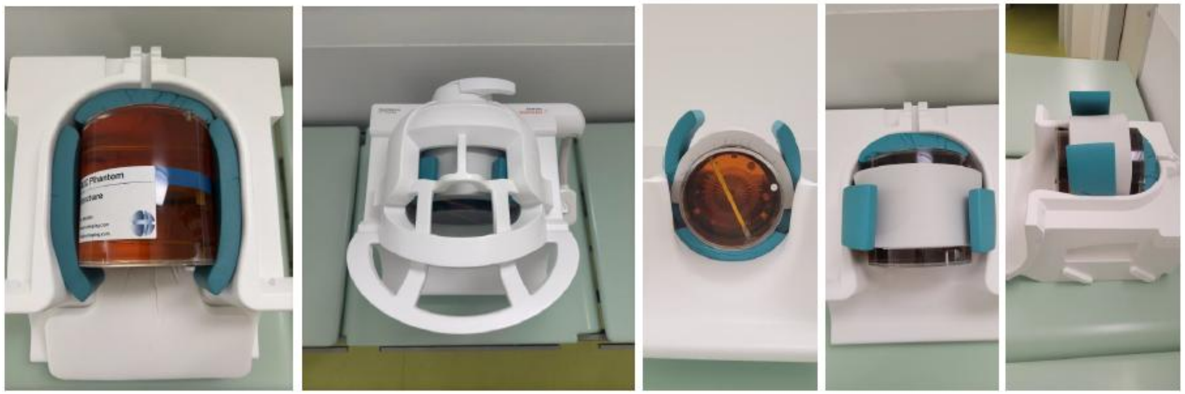

Document S2

Accompanying information for the raters. *Within this manuscript, the structure of the phantom was explained in detail both schematically and with the help of pictorial examples for the raters. In addition, a detailed list of negative and positive examples of the respective criteria to be evaluated was provided. This approach was intended to train the raters in order to optimally prepare them for the qualitative assessment of the images using the HON tool.*

Structure of the DWI Model

The cylindrical model used in this study to investigate retinal ischemia simulates tissue with different diffusion properties, represented by subsequent apparent diffusion coefficient (ADC) values. The compartment labeled as "ADC_{RD}" in the figure below has an ADC value of 0.5×10^{-3} mm^2/s, which is characteristic of cytotoxic edema in the central nervous system (CNS). This compartment represents the region with restricted extracellular diffusion. In contrast, the compartment labeled as "ADC_{GM}" exhibits an ADC value of 0.8×10^{-3} mm^2/s, corresponding to the average ADC value of healthy neuronal tissue (gray matter, GM).

For the subsequent evaluation of the generated images, both the lesion insertion and the resolution assessment are of key importance. The lesion insertion simulates diffusion-restricted foci (ADC value of 0.5×10^{-3} mm^2/s) of decreasing size (see right figure), embedded within the compartment representing healthy neuronal tissue (ADC value of 0.8×10^{-3} mm^2/s). At the end of the model, a Siemens star is incorporated to facilitate a comparison of the resolution capabilities of the respective sequences.

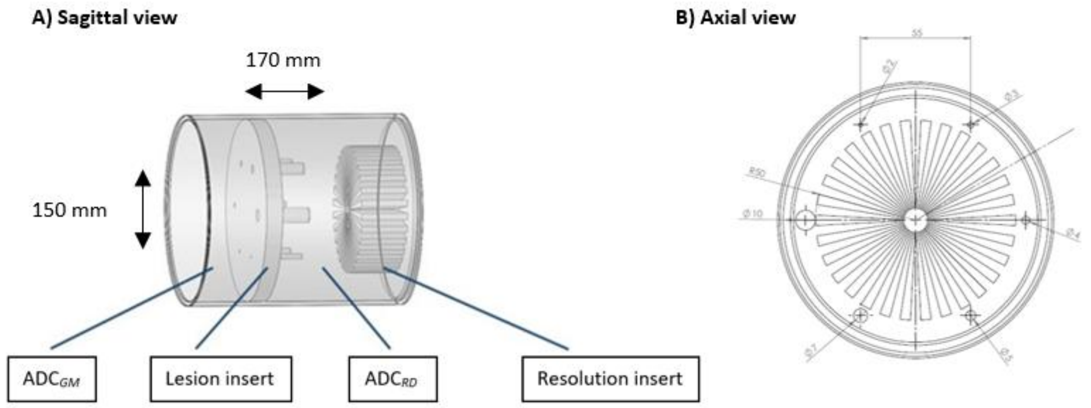

source: Image © HQ Imaging, modified by the authors. Used with permission of Dr. Michael Bach.

To establish a basis for the subsequent evaluation of the acquired images and to provide an initial understanding of the model, the following pages first present the model structure in T2-weighted and B-1000-weighted imaging, along with both a positive and a negative example in B-1000-weighting for each evaluation category.

Please familiarize yourself with the respective sections and the specific details that should be considered. Since only the region between the lesion insertion and the Siemens star is relevant for the qualitative comparison of the images, the images in HON have also been restricted to this section. However, to facilitate an understanding of the model, an image example is first provided for each compartment marked in the figure. The order of the images from top to bottom corresponds to the layers of the HON acquisitions.

Image Examples of the DWI Model Structure

Compartment	T2-weighting	B-1000-weighting	ADC map
gray matter (GM)<br><br>ADC value: 0.8 x 10^{-3} mm²/s	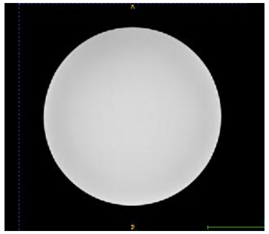	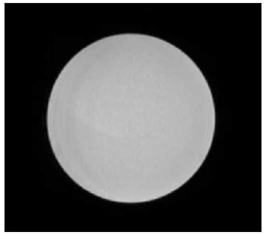	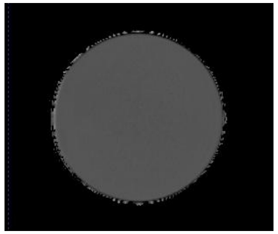
ischemic lesions with restricted extracellular diffusion (RD) embedded in gray matter	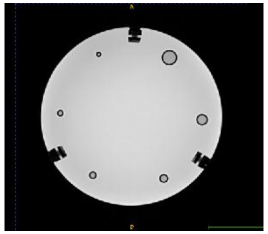	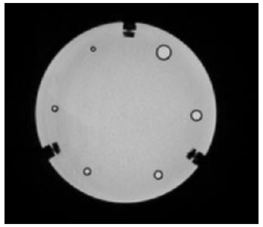	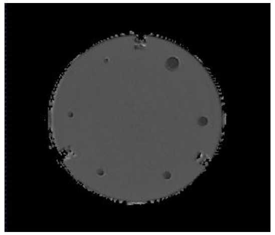
Siemens Star	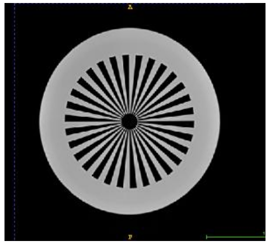	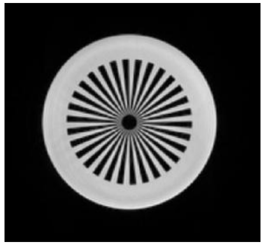	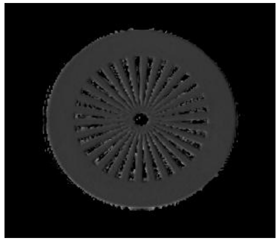
restricted extracellular diffusion (RD)<br><br>ADC value: 0.5 x 10^{-3} mm²/s	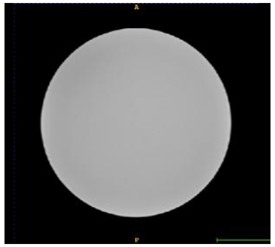	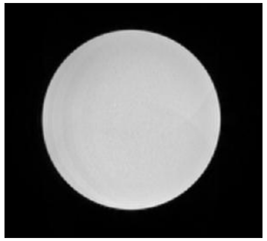	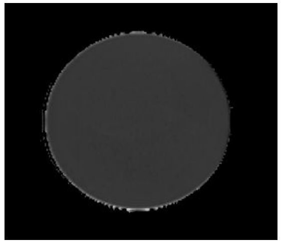

Image examples for evaluation criteria

Criteria	Positive example	Negative example
Lesion Visibility / Contrast<br><br>In the **positive example**, lesions are well distinguishable from the surrounding matrix. The **negative example** shows poor contrast, making it difficult to differentiate the lesion from the background tissue. The visibility of the lesion strongly depends on **windowing settings.**	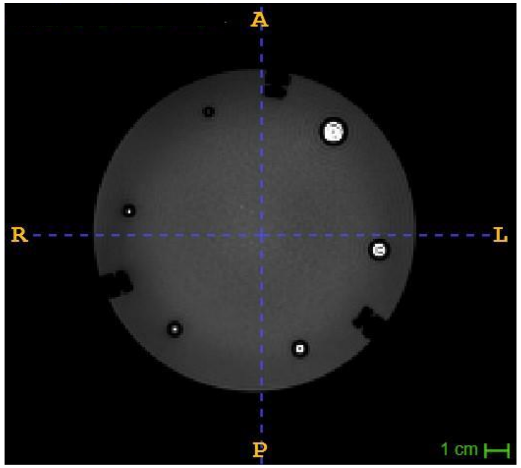	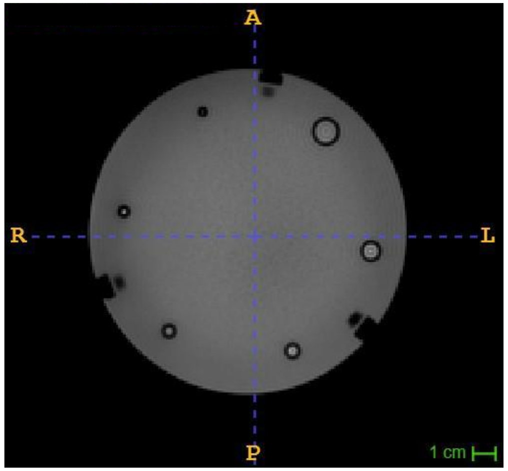
Susceptibility Artifacts<br><br>In the **negative example**, a **hyper intense susceptibility artifact** is visible at the interface of the materials used in the model	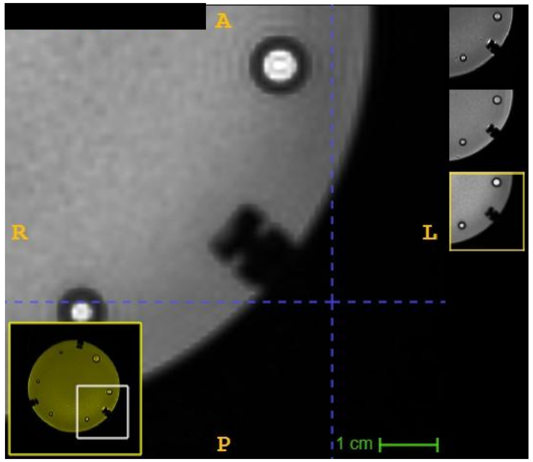	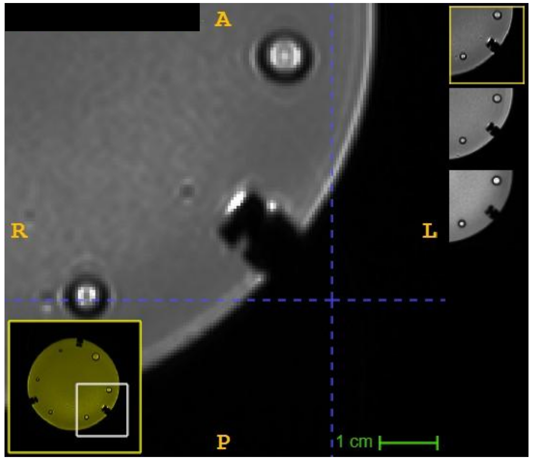

Geometric Distortion<br><br>In the **negative example**, an increased **edge blurring** of the Siemens star structures is subjectively noticeable. Additionally, the structures appear **reduced in size**, indicating geometric distortions.	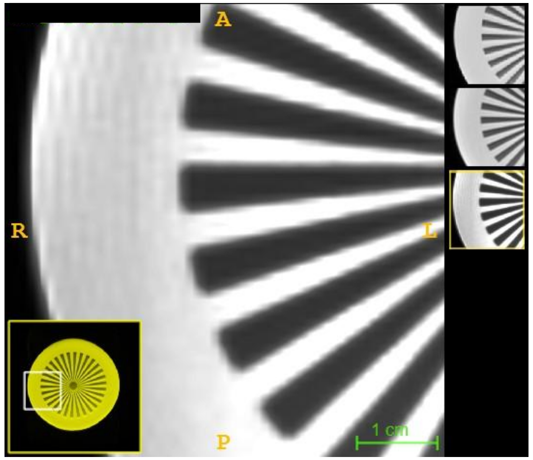	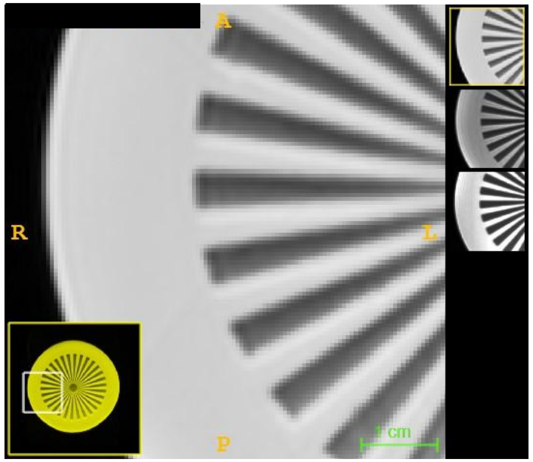
Image Noise<br><br>In the **negative example**, the image appears subjectively **more irregular** and exhibits a **higher level of noise**.	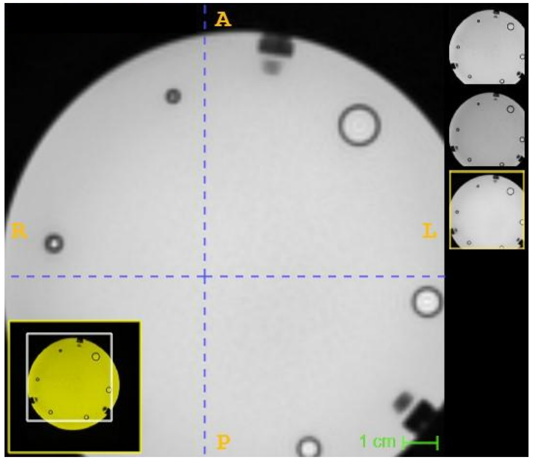	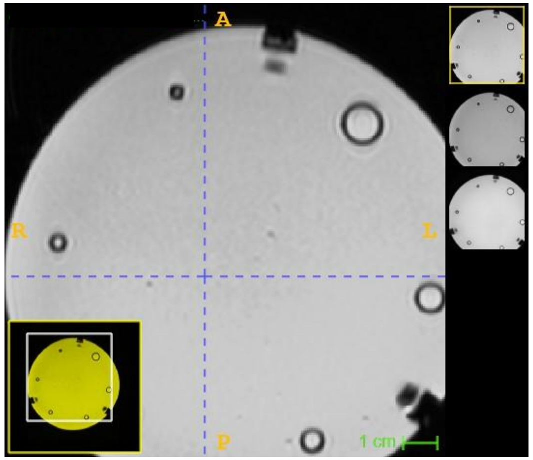

Image Resolution<br><br>In the **positive example**, the Siemens star appears **better resolved**, with clearer delineation of the radial structures. This improved resolution allows for a more precise identification of structural details and better separation between adjacent features.	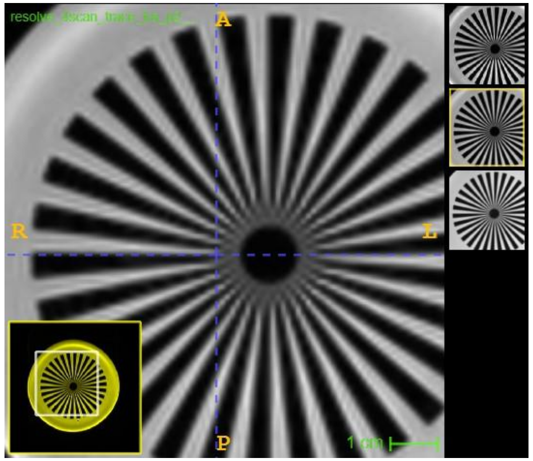	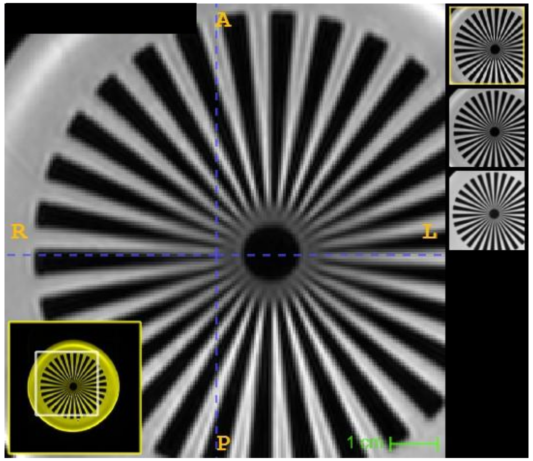
Overall Image Quality<br><br>In the **positive example**, the overall image quality appears **more cohesive**, with **better edge sharpness**, a **more homogeneous base structure**, and a clearer delineation of the lesions from the surrounding tissue. Additionally, the **hypo intense edges of the lesions** are **sharply separated** from the matrix.	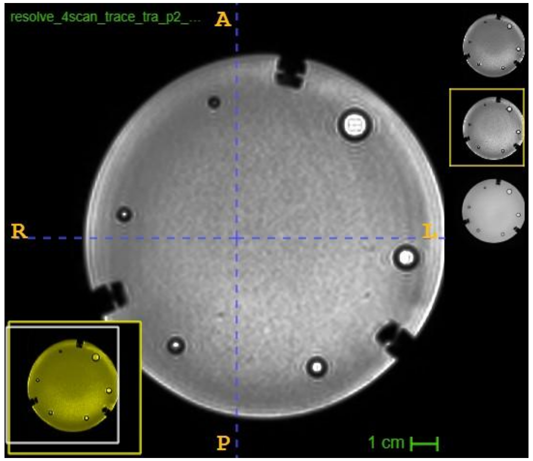	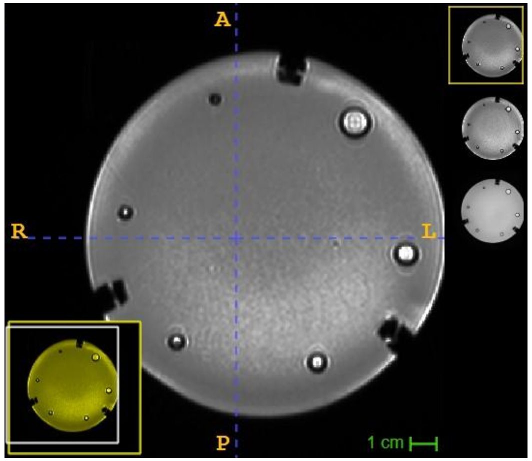

After familiarizing yourself with the model and evaluation criteria, please proceed with study 186, titled *“pre study: ss-EPI, rs-EPI (RESOLVE), and BLADE DWI in the diagnostic performance of retinal ischemia”* in HON. There, you can apply the background information you have gained to three image examples, which are labeled in descending order of quality as "good," "medium," and "poor."

Figure S3

Comparison of all quantitative parameters including the 2 mm lesion. *Due to the small lesion size of 2 mm and the selected voxel volume (0.5 x 0.5 x 3 mm), systematic differences in measured values are evident, particularly with regard to ADC, CNR, ReCon, and also GDR. Due to this measurement distortion, statistical comparisons were not calculated in this plot.*

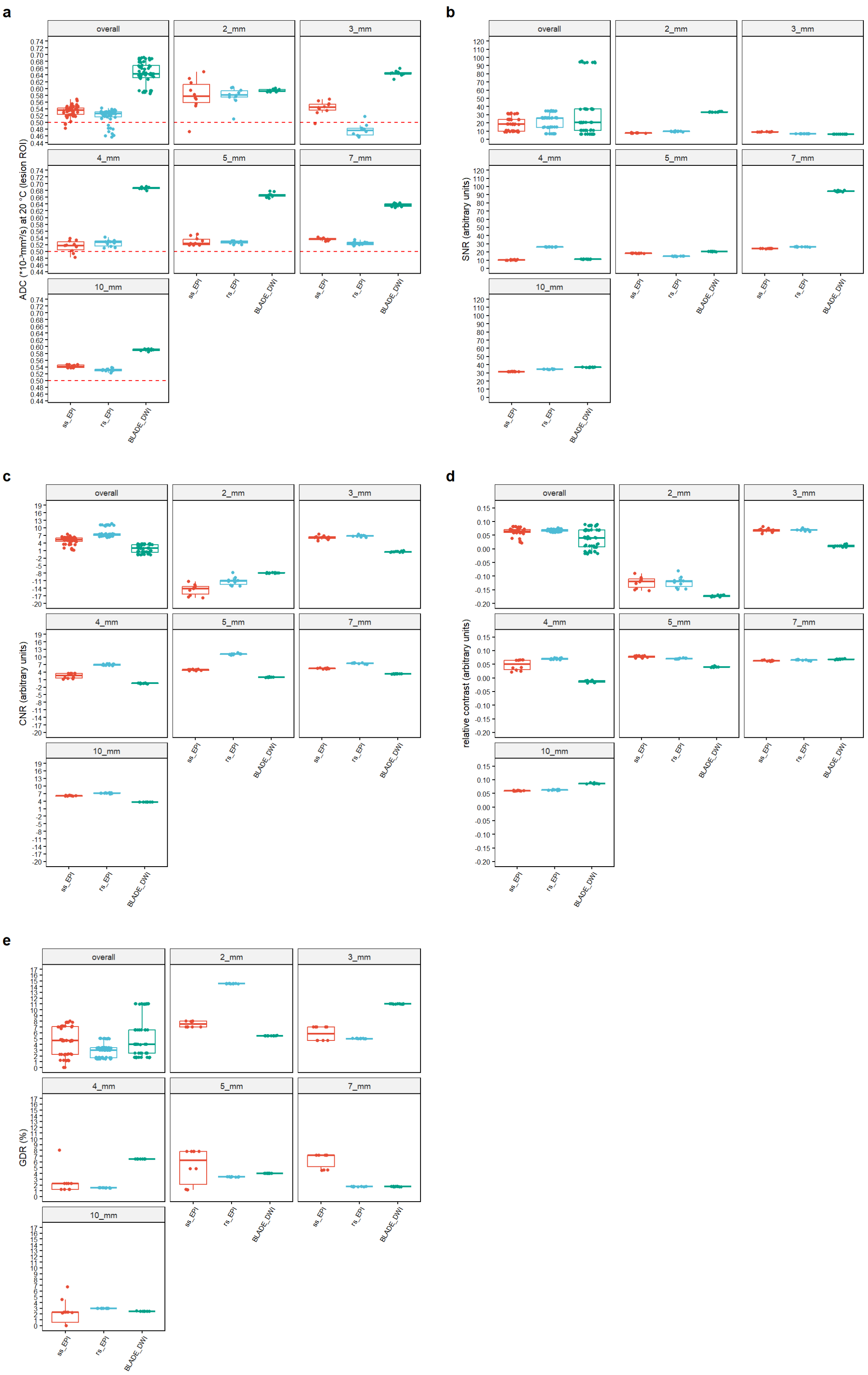

Table S4

Quantitative metrics of the three sequences. *Values are displayed as mean ± SD (Min./Max.) and arranged in order of lesion size from top to bottom: total value, 10 mm, 7 mm, 5 mm, 4 mm, 3 mm, 2 mm. The values of the 2 mm lesion are highlighted in red. Together with Figure S3, it can be seen that the CNR, ReCon, and GDR values in particular are significantly altered compared to the other lesion sizes. The reduced signal detected in the ROI not only affects the CNR and ReCon values, but also causes a significant distortion of the ADC values, especially for the EPI sequences, compared to the other lesion sizes. Since the measured values depend on various factors, such as PVE or PSF, pixel size, or crosstalk due to the selected layer spacing, interpretation is difficult, and we therefore decided not to include these values in the main analysis in the paper.*

	ss-EPI-DWI	rs-EPI-DWI	TGSE-BLADE-DWI
$ADC_{20°}$	0.53 ± 0.02 (0.48 – 0.57)	0.52± 0.02 (0.46 – 0.54)	0.65 ± 0.03 (0.58 – 0.69)
	0.54 ± 0.004 (0.54 – 0.55)	0.53 ± 0.005 (0.52 – 0.54)	0.59 ± 0.002 (0.58 – 0.59)
	0.54 ± 0.004 (0.53 – 0.54)	0.52 ± 0.01 (0.52 – 0.53)	0.64 ± 0.004 (0.63 – 0.64)
	0.53 ± 0.01 (0.52 – 0.55)	0.53 ± 0.005 (0.52 – 0.53)	0.67 ± 0.01 (0.66 – 0.68)
	0.52 ± 0.02 (0.48 – 0.54)	0.52 ± 0.01 (0.51 – 0.54)	0.69 ± 0.004 (0.68 – 0.69)
	0.54 ± 0.02 (0.5 – 0.57)	0.48 ± 0.02 (0.46 – 0.52)	0.64 ± 0.01 (0.63 – 0.66)
	0.58 ± 0.05 (0.47 – 0.65)	0.58 ± 0.03 (0.51 – 0.60)	0.59 ± 0.004 (0.59 – 0.60)
**SNR**	18.58 ± 8.55 (8.69 – 31.74)	21.65 ± 9.78 (6.76 – 34.81)	33.74 ± 32.33 (6.07 – 95.24)
	31.42 ± 0.23 (31.14 – 31.74)	34.41 ± 0.12 (34.19 – 34.81)	36.82 ± 0.18 (36.62 – 37.16)
	24.01 ± 0.06 (23.92 – 24.12)	26.19 ± 0.2 (25.89 – 26.54)	94.21 ± 0.75 (93.15 – 95.24)
	18.32 ± 0.2 (18.0 – 18.52)	14.78 ± 0.14 (14.63 – 15.01)	20.46 ± 0.14 (20.18 – 20.59)
	10.16 ± 0.44 (9.61 – 10.62)	26.02 ± 0.2 (25.71 – 26.34)	11.06 ± 0.04 (10.99 – 11.11)
	8.97 ± 0.18 (8.69 – 9.23)	6.87 ± 0.07 (6.76 – 6.96)	6.12 ± 0.04 (6.07 – 6.23)
	7.63 ± 0.35 (7.19 – 8.34)	9.51 ± 0.46 (8.93 – 10.42)	33.37 ± 0.37 (33.03 – 34.12)
**CNR**	4.98 ± 1.48 (1.07 – 7.49)	7.87 ± 1.66 (6.19 – 11.65)	1.76 ± 1.59 (-0.8 – 3.67)
	6.12 ± 0.11 (5.99 – 6.31)	7.15 ± 0.15 (6.87 – 7.32)	3.58 ± 0.05 (3.52 – 3.67)
	5.39 ± 0.17 (5.09 – 5.61)	7.43 ± 0.21 (6.99 – 7.65)	3.24 ± 0.06 (3.12 – 3.35)
	4.82 ± 0.25 (4.38 – 5.09)	11.09 ± 0.26 (10.69 – 11.65)	1.95 ± 0.1 (1.82 – 2.13)
	2.46 ± 1.05 (1.07 – 3.48)	6.91 ± 0.31 (6.43 – 7.27)	-0.53 ± 0.15 (-0.8 - -0.35)
	6.12 ± 0.73 (4.87 – 7.49)	6.79 ± 0.4 (6.19 – 7.53)	0.53 ± 0.22 (0.25 – 0.89)
	-14.59 ± 2.15 (-17.73 - -11.3)	-11.19 ± 1.66 (-13.19 - -7.73)	-7.93 ± 0.12 (-8.09 - -7.71)
**ReCon**	0.06 ± 0.01 (0.02 – 0.08)	0.07 ± 0.004 (0.06 – 0.08)	0.04 ± 0.04 (-0.02 – 0.09)
	0.06 ± 0.001 (0.06 – 0.06)	0.06 ± 0.001 (0.06 – 0.06)	0.09 ± 0.002 (0.08 – 0.09)
	0.06 ± 0.002 (0.06 – 0.07)	0.07 ± 0.002 (0.06 – 0.07)	0.07 ± 0.001 (0.07 – 0.07)
	0.08 ± 0.004 (0.07 – 0.08)	0.07 ± 0.001 (0.07 – 0.07)	0.04 ± 0.002 (0.04 – 0.04)
	0.05 ± 0.02 (0.02 – 0.07)	0.07 ± 0.003 (0.07 – 0.07)	-0.01 ± 0.003 (-0.02 - -0.01)
	0.07 ± 0.01 (0.06 – 0.08)	0.07 ± 0.004 (0.06 – 0.08)	0.01 ± 0.004 (0.01 – 0.02)
	-0.12 ± 0.02 (-0.15 – 0.09)	-0.12 ± 0.02 (-0.15 - -0.08)	-0.17 ± 0.003 (-0.18 - -0.17)
**GDR**	4.42 ± 2.62 (0 – 8)	2.92 ± 1.28 (1.5 – 5)	5.14 ± 3.39 (1.71 – 11)
	2.26 ± 2.11 (0 – 6.7)	3 ± 0 (3 – 3)	2.5 ± 0 (2.5 – 2.5)
	6.37 ± 1.24 (4.57 – 7.14)	1.71 ± 0 (1.71 – 1.71)	1.71 ± 0 (1.71 – 1.71)
	5.22 ± 3.02 (1.2 – 7.8)	3.4 ± 0 (3.4 – 3.4)	4 ± 0 (4 – 4)
	2.43 ± 2.02 (1.25 – 8)	1.5 ± 0 (1.5 – 1.5)	6.5 ± 0 (6.5 – 6.5)
	5.83 ± 1.23 (4.67 – 7)	5 ± 0 (5 – 5)	11 ± 0 (11 – 11)
	7.5 ± 0.53 (7 – 8)	14.5 ± 0 (14.5 – 14.5)	5.5 ± 0 (5.5 – 5.5)
